# Comprehensive evaluation of molecular enhancers of the isothermal exponential amplification reaction

**DOI:** 10.1038/srep37837

**Published:** 2016-12-02

**Authors:** Ellie Mok, Eugene Wee, Yuling Wang, Matt Trau

**Affiliations:** 1 Centre for Personalized Nanomedicine, Australian Institute for Bioengineering and Nanotechnology (AIBN), Corner College and Cooper Roads (Bldg 75), Brisbane, QLD 4072, Australia; 2 School of Chemistry and Molecular Biosciences, The University of Queensland, Brisbane, QLD 4072, Australia

## Abstract

The exponential amplification reaction (EXPAR) is an emerging isothermal nucleic acid amplification method with high potential for molecular diagnostics due to its isothermal nature and high amplification efficiency. However, the use of EXPAR is limited by the high levels of non-specific amplification. Hence, methods that can improve the specificity of EXPAR are desired to facilitate its widespread adoption in practice. Herein, we proposed a strategy to improve EXPAR performance by using molecular enhancers. Eight small molecules were investigated, including ethylene glycol, propylene glycol, betaine, dimethyl sulfoxide (DMSO), trehalose, tetramethylammonium chloride (TMAC), bovine serum albumin (BSA) and single-stranded binding (SSB) proteins. A combination of kinetic and end-point analysis was adopted to investigate how these molecules affected EXPAR performance. Trehalose, TMAC, BSA and SSB proteins were found to have positive effects on EXPAR with trehalose being able to increase the efficiency of EXPAR. In contrast, TMAC, BSA and SSB proteins were shown to increase the specificity of EXPAR. We applied our findings to demonstrate the combination of trehalose and TMAC could simultaneously improve both the efficiency and specificity of an EXPAR-based miRNA detection method. The information provided in this study may serve as a reference to benefit the wider isothermal amplification community.

Isothermal exponential amplification reaction (EXPAR) is an emerging amplification technique that is used to amplify short oligonucleotides for molecular diagnostics^1^. In contrast with the conventional PCR, EXPAR operates at a constant temperature and provides high amplification efficiency, allowing 10^6^–10^9^ fold amplification of short oligonucleotides within minutes^2^,^3^,^4^. Owing to its advantages, many studies have reported the use of EXPAR for molecular diagnostics^5^,^6^,^7^.

Briefly, EXPAR amplifies DNA by four major steps (Fig. 1). First, EXPAR is initiated when the target primes to the trigger sequence of the template, forming a partial double-stranded duplex. This is followed by the extension by DNA polymerase, forming an extended double-stranded DNA containing a nicking enzyme recognition site. A nicking enzyme then cleaves the upper strand and DNA polymerase displaces the cleaved trigger by strand displacement to generate additional trigger sequences. This occurs repeatedly and exponentially^1^.

Despite its advantages, target-independent amplification is a major limitation of EXPAR, which in turn, leads to lowered specificity and sensitivity^1^,^5^,^8^. Non-specific amplification of EXPAR was suggested to arise from the interaction between the single-stranded template and the DNA polymerase independent of the target^3^. Due to the issue of high levels of non-specific amplification with EXPAR, several studies have proposed ways to resolve this problem. Since the non-specific amplification is sequence dependent, Qian *et al*. demonstrated that the variability in template performance is linked to sequence motifs^9^. Therefore, they developed computational methods to predict EXPAR template performance based on the template sequence. Wang *et al*. described the use of graphene oxide (GO) to minimize the level of non-specific amplification by preventing the binding of the DNA polymerase to the template^10^. In the presence of a target, the template sequestered by GO can then be released, initiating EXPAR. This thus led us to hypothesize that other small molecules could also be used to enhance EXPAR performance.

Small molecules have been shown to improve the PCR amplification^11^,^12^,^13^,^14^,^15^,^16^,^17^,^18^, however, the use of these small molecules has not been described in detail for EXPAR, nor have the effects of these small molecules on EXPAR been studied. Understanding the effects of these small molecules on EXPAR can potentially facilitate applications in diagnostics.

In this study, we investigated the effects of several small molecules including ethylene glycol, propylene glycol, betaine, DMSO, trehalose, TMAC, BSA and SSB proteins on EXPAR. We adopted a combination of kinetic and end-point analysis to investigate the effects of these molecules as EXPAR additives. It was found that trehalose, TMAC, BSA and SSB proteins had positive effects on EXPAR with trehalose being able to increase the efficiency of EXPAR, while TMAC, BSA and SSB proteins were found to increase the specificity of EXPAR. The increased efficiency of EXPAR in the presence of trehalose could be due to its role in reducing the melting temperature (Tm) of the template, and stabilizing enzymes in the reactions^13^,^19^. In contrast, the improvement of specificity observed in the presence TMAC, BSA and SSB proteins could be due to their role in reducing potential DNA/RNA mismatch or relieving interference^16^,^17^,^18^. Finally, we applied our findings to improve on an EXPAR-based miRNA detection method. It was found that the combination of trehalose and TMAC could synergistically improve the efficiency and specificity of an EXPAR-based miRNA detection method. We believe our data could be extended to other isothermal amplification systems to improve their analytical performances in a wide range of applications.

## Results and Discussion

### Study design

**Figure 1:**
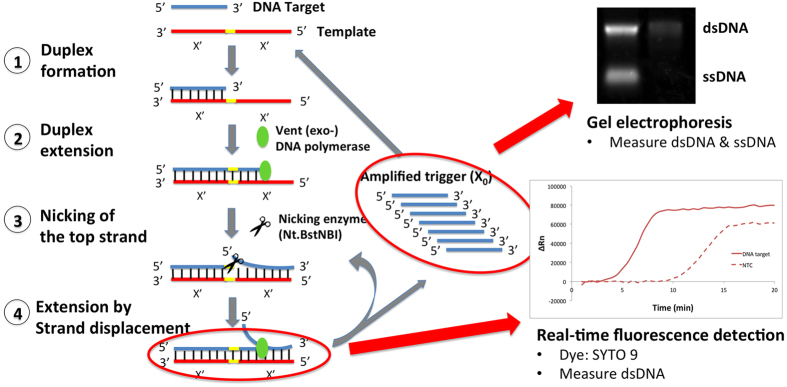
The principle of the EXPAR and general strategy adopted.

The effects of several small molecules on EXPAR were studied in this work to investigate their potential to enhance EXPAR by increasing reaction efficiency (defined as product yield and rate of product generation) and specificity (defined as differences in signal between target and target-independent amplification). As shown in Fig. 1, EXPAR generates both double-stranded DNA (dsDNA) and single-stranded DNA (ssDNA)^1^. As we were interested in the rate of generation and yield of both double-stranded and single-stranded products, two complementary approaches (kinetic and end-point measurements) were employed (Fig. 1). To assess the rate for product generation, we performed real-time kinetic measurements of fluorescence accumulation from a dsDNA specific dye as dsDNA species increased over the duration of the EXPAR reaction. Using this technique, one could evaluate changes in EXPAR efficiency (defined by the speed of which a detectable signal was observed) and specificity (defined by the reaction time lag between target and target-independent amplification). Then, to evaluate both the yield (defined as the amount of amplified DNA) and specificity (defined as the differences in DNA yield between target and target-independent amplifications) of EXPAR products and also to validate data from real time measurements, end-point gel electrophoresis analysis was employed.

### Trehalose increased EXPAR yield

The effect of trehalose was investigated because of its ability to lower DNA Tm and thermostabilize enzymes^13^,^19^, and therefore could be beneficial in improving specificity and yield in situations where the working reaction temperature was not optimal for enzymatic function. For example, EXPAR is typically performed at a temperature higher than the optimal temperature of the nicking enzyme.

**Figure 2:**
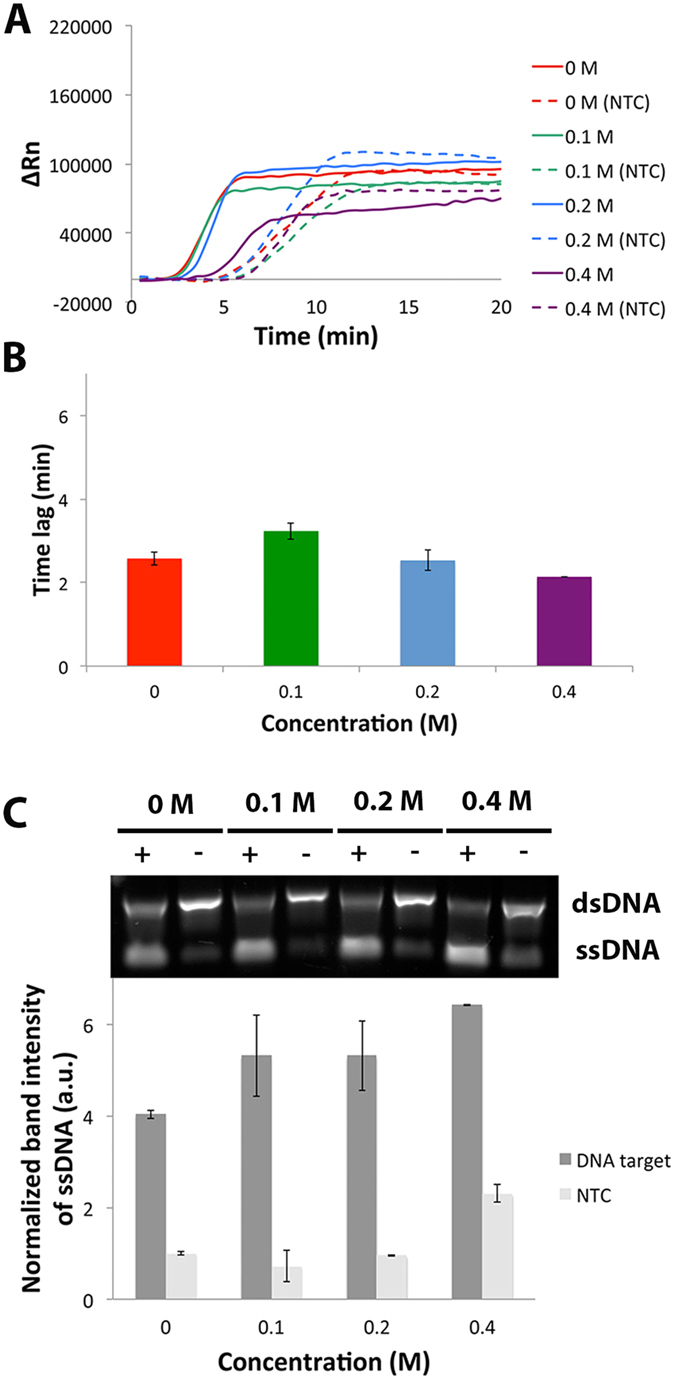
Effects of different concentrations of trehalose on EXPAR. (**A**) Typical real-time amplification plot of EXPAR performed with positive target (solid line) and no-target control (NTC, dotted lines) at 0, 0.1, 0.2 and 0.4 M of trehalose. (**B**) Time lag between targeted and NTC at different concentrations of trehalose. (**C**) Top: Typical gel electrophoresis image of corresponding EXPAR products. Full-length gel image provided in Supplementary Figure S8. Bottom: Bar graph of average ssDNA band intensities normalized to NTC without trehalose. Error bars represent SD, n = 2.

The effects of different concentrations of trehalose on EXPAR are shown in Fig. 2. From the real time experiments, trehalose did not seem to affect the rate of EXPAR at 0, 0.1 and 0.2 M as these reactions were detected within a similar time frame. However, the rate was decreased at 0.4 M as evidenced by the delay of reaction (Fig. 2A). This could be due to two reasons. First, high concentration of trehalose results in a large reduction of Tm between target and template (see Supplementary Table S1), which could affect the hybridization efficiency thus EXPAR rates. Second, high concnetration of trehalose could interact with itself to form macromolecular clusters, acting as a crowding agent^20^. This could decrease diffusion rates, and therefore affect the rate of amplification. Trehalose only modestly improved specificity at 0.1 M as evidenced by the increased lag between the targeted and non-targeted reactions (Fig. 2B). However, at higher amounts of trehalose, this benefit was reversed. From the end-point data (Fig. 2C), trehalose dramatically improved EXPAR ssDNA yield as evidenced by the higher ssDNA band intensities. However, no improvement in specificity was observed. In fact, at high amounts of trehalose, target-independent amplification increased.

### TMAC, BSA and SSB proteins increased specificity of EXPAR

TMAC has the ability to alter the Tm of DNA and eliminate the usual dependence of the DNA transition temperature on base composition^16^,^17^,^21^,^22^,^23^, hence the use of TMAC in EXPAR could be also beneficial in improving specificity.

**Figure 3:**
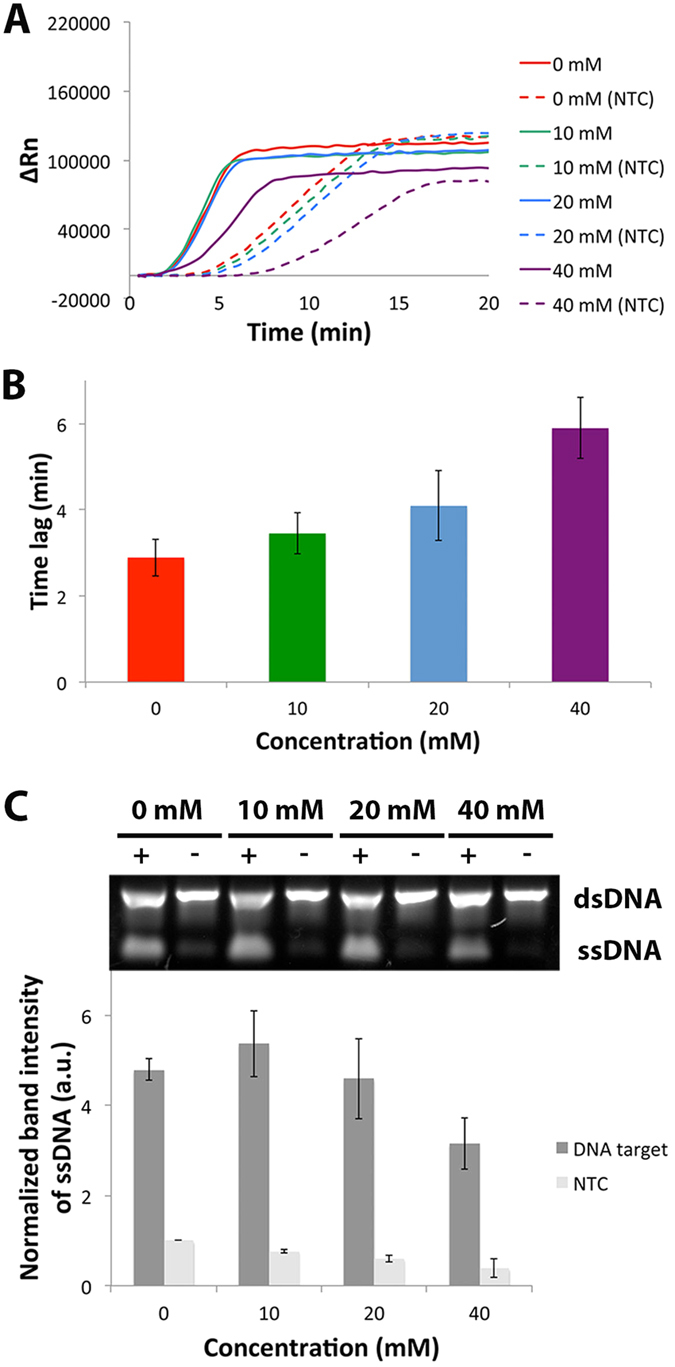
Effects of different concentrations of TMAC on EXPAR. **(A**) Typical real-time amplification plot of EXPAR performed with positive target (solid line) and no-target control (NTC, dotted lines) at 0, 10, 20 and 40 mM of TMAC. (**B**) Time lag between targeted and NTC at different concentrations of TMAC. (**C**) Top: Typical gel electrophoresis image of corresponding EXPAR products. Full-length gel image provided in Supplementary Figure S8. Bottom: Bar graph of average ssDNA band intensities normalized to NTC without TMAC. Error bars represent SD, n = 2.

The effects of different concentrations of TMAC on EXPAR are shown in Fig. 3. From the real-time experiments, TMAC did not seem to affect the rate of EXPAR at 0, 10 and 20 mM as these reactions were detected within a similar time frame. However, a decreased rate was observed at 40 mM as evidenced by the delayed start time of the reaction (Fig. 3A). However, TMAC dramatically improved specificity at 40 mM as evidenced by the increased lag between the targeted and non-targeted reactions (Fig. 3B). From the end-point data (Fig. 3C), TMAC markedly improved specificity at 40 mM as evidenced by the 50% reduction of ssDNA band intensities for the non-targeted reaction, consistent with the real time data.

Apart from TMAC, BSA and SSB proteins were also able to increase the specificity of PCR by relieving interference^16^,^18^,^24^,^25^. BSA was found to relieve inhibition from samples sequestering inhibitors and stabilizing Taq DNA polymerase^26^,^27^, while SSB proteins bind non-specifically to single-stranded products and act to stabilize single-stranded products^28^. Therefore, BSA and SSB proteins could also be beneficial in improving specificity.

The effects of different concentrations of BSA on EXPAR are shown in Supplementary Figure S1. From the real-time experiments, BSA did not seem to affect the rate of EXPAR as all target-dependent amplification were detected within a similar time frame for all amounts of BSA added. BSA did not improve specificity as evidenced by the decreased lag between the target and non-targeted reactions. However, from the end-point data, 40 mg/mL of BSA dramatically improved specificity as evidenced by the lower ssDNA band intensities (i.e. 0.27 fold reduction) for the no-target control (NTC).

The effects of SSB proteins on EXPAR are shown in Supplementary Figure S2. From the real-time experiments, SSB proteins did not seem to affect the rate of EXPAR at 0 and 5 μg/mL as these reactions were detected within a similar time frame. However, the rate was decreased at 7.5 and 10 μg/mL as evidenced by the delayed start time of reactions. However, SSB proteins notably improved specificity as evidenced by the increased lag between the targeted and non-targeted reactions. From the end-point data, SSB proteins dramatically improved specificity at 10 μg/mL as evidenced by the lower ssDNA band intensity (i.e. 0.28 fold reduction) for NTC, consistent with the real time data. Finally, only moderate improvement in yield was observed at 5 μg/mL and 7.5 μg/mL of SSB proteins.

The addition of TMAC, BSA and SSB proteins decreased the generation of non-specific products in different ways (Fig. 3, see Supplementary Figures S1 and S2). However, the molecular mechanism of the enhancing effect of these small molecules in nucleic acid amplification is not well understood. The ability of TMAC to increase specificity in EXPAR could be a result of reducing potential DNA mismatch, and thermal normalization of AT and GC base pairings^16^. The increased specificity of EXPAR by BSA could be due to its ability to scavenge inhibitors^25^. One possible mechanism of SSB proteins in EXPAR is that SSB proteins could bind to the single-stranded motifs of mismatched based pairings, thus sterically preventing further non-specific amplification^29^.

### Ethylene glycol, propylene glycol, betaine and DMSO decreased yield of EXPAR

Other additives that generally act to lower DNA Tm were also tested on EXPAR: ethylene glycol, propylene glycol, betaine and DMSO. Betaine, an isostabilizing agent, can eliminate base pair composition-dependence of DNA thermal melting transition^15^. DMSO lowers DNA Tm and disrupts base pairing and facilitates strand separation by interfering with hydrogen bonding^14^,^30^. Due to the functions of these small molecules, they could be beneficial in improving specificity and yield of EXPAR.

The effects of ethylene glycol on EXPAR are shown in Supplementary Figure S3. From the real-time experiments, ethylene glycol did not seem to affect the rate of EXPAR as all target-dependent amplification were detected within a similar time frame for 0, 0.9 and 0.18 M of ethylene glycol added. No amplification was observed with 0.36 M of ethylene glycol added. Ethylene glycol did not seem to improve specificity as evidenced by the similar time lag between the targeted and non-targeted reactions. From the end-point data, ethylene glycol improved specificity at 0.9 M and 0.18 M. This improvement in specificity was at the expense of ssDNA amplification yield as evidenced by the lower ssDNA band intensities.

Very similar results were obtained for propylene glycol, betaine and DMSO in which higher concentration of these small molecules increased specificity (see Supplementary Figures S4, S5 and S6). However, they dramatically decreased yield. The effects of betaine on EXPAR are shown in Supplementary Figure S5. From the real-time experiments, betaine seemed to affect the rate of EXPAR as the higher the concentration of betaine was, the later the reaction was detected. No amplification was observed with 2.5 M of betaine added. Betaine improved specificity at 0.5 M and 1 M as evidenced by the increased lag between the targeted and non-targeted reactions. From the end-point data, betaine improved specificity at 0.5 mg/mL and 1 mg/mL. However, betaine decreased EXPAR ssDNA yield as evidenced by the lower ssDNA band intensities.

Ethylene glycol, betaine, DMSO and propylene glycol were expected to increase the efficiency of EXPAR based on their roles in reducing DNA Tm. However, these molecules were instead found to decrease the efficiency of EXPAR (see Supplementary Figures S3, S4, S5 and S6). A possible explanation was that the system was already at its optimal temperature, hence further decreasing DNA Tm would instead prevent the target from hybridizing with the template.

Although trehalose has the same role as ethylene glycol, betine, DMSO and propylene glycol in reducing DNA Tm, increased in yield was observed for reactions that contained trehalose (Fig. 2). Our hypothesis is that the observed increased yield was due to its role in thermostabilizing enzymes. The nicking enzyme N.BstNBI in EXPAR operates from 37 °C to 65 °C, with an optimum temperature of 55 °C^31^. Since EXPAR operates at 60 °C, which was above optimum temperature for the nicking enzyme, it may be possible that trehalose was able to stabilize N.BstNBI, and thus improved its activity which in turn, resulted in higher ssDNA yields (Fig. 2C).

In brief, the addition of some small molecules can enhance the performance of EXPAR. A summary of our findings is provided in Table 1.

### Combination of trehalose and TMAC increased both yield and specificity of EXPAR

High efficiency and specificity are desired for the amplification of nucleic acids. Since trehalose could increase yield while TMAC could increase the specificity of EXPAR, we wondered if the combination of trehalose and TMAC could have a synergistic benefit.

**Figure 4:**
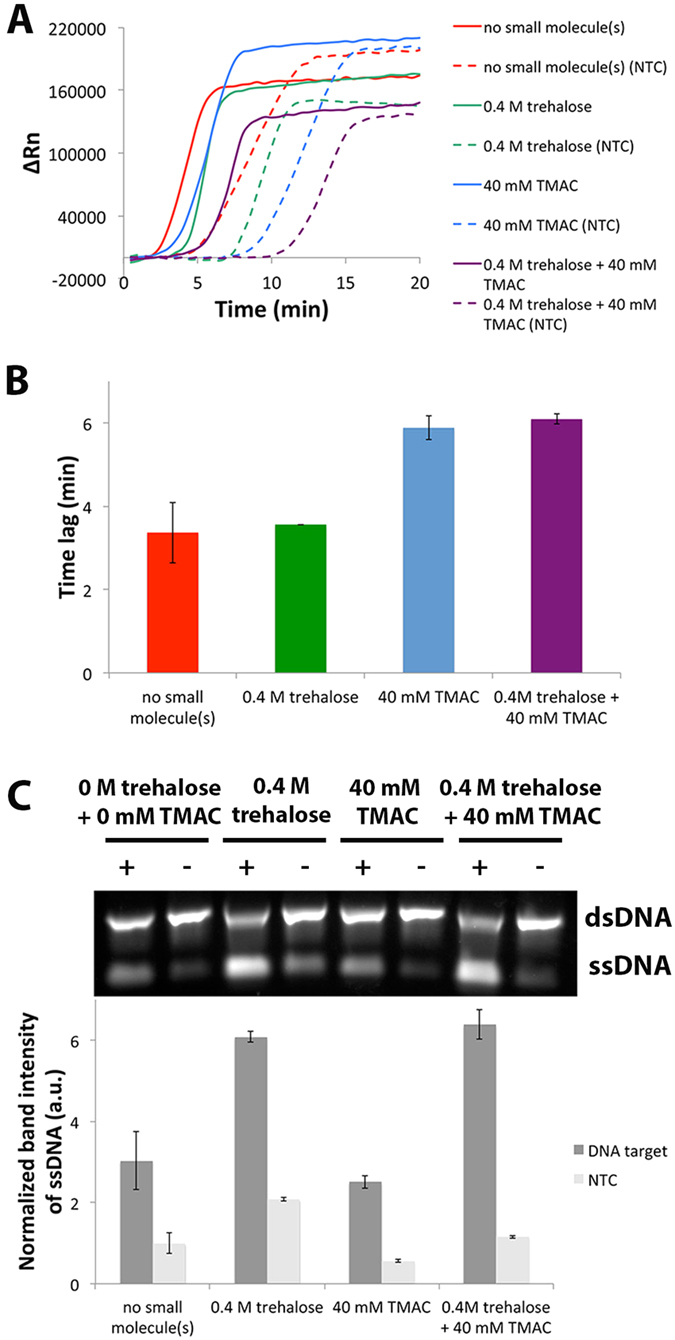
Effects of combining trehalose and TMAC on EXPAR. (**A**) Typical real-time amplification plot of EXPAR performed with positive target (solid line) and no-target control (NTC, dotted lines) at 0 M of trehalose +0 mM of TMAC, 0.4 M trehalose, 40 mM TMAC and 0.4 M trehalose +40 mM TMAC. (**B**) Time lag between targeted and NTC with or without TMAC and trehalose. (**C**) Top: Typical gel electrophoresis image of corresponding EXPAR products. Full-length gel image provided in Supplementary Figure S8. Bottom: Bar graph of average ssDNA band intensities normalized to NTC without trehalose and TMAC. Error bars represent SD, n = 2.

The effects of the combination of trehalose and TMAC on EXPAR are shown in Fig. 4. From the real time experiments, the combination of trehalose and TMAC did not seem to affect the rate of EXPAR over the individual additives as all target-dependent amplification were detected within a similar time frame (Fig. 4A). However, the combination of trehalose and TMAC distinctly improved specificity similar to TMAC alone as evidenced by the increased lag between the targeted and non-targeted reactions (Fig. 4B). In addition, from the end-point data (Fig. 4C), the combination of trehalose and TMAC significantly improved EXPAR ssDNA yield similar to that of trehalose alone while maintaining specificity.

A DNA melt analysis was also performed to further understand how the small molecules affected EXPAR (Supplementary Table S1). Briefly, under current EXPAR conditions, 1.8 M ethylene glycol, 0.8 M propylene glycol, 1 M betaine, 5% DMSO, 0.4 M trehalose and 40 mM TMAC lowered the Tm between target and template by at least 2 °C. Each of those small molecules decreased Tm to different degrees, consistent with the literature^15^,^22^,^32^,^33^,^34^,^35^. In contrast, 1 mg/mL BSA and 10 μg/mL SSB proteins had minimal effect on Tm (1 °C or less).

### Improvements to EXPAR-based assays and other isothermal applications

Due to the positive effects of trehalose and TMAC on EXPAR, we were interested in translating our findings to a potential application. To this end, we designed an EXPAR-based assay for detecting miRNA analogues followed by a surface-enhanced Raman spectroscopy (SERS) visualization to evaluate the improvement in analytical performance. SERS is a vibrational readout technique that has experienced significant growth recently due to its capability to selectively and sensitively detect biomolecules. It is based on enhanced Raman scattering when molecules are adsorbed on rough metal surfaces^36^.

**Figure 5:**
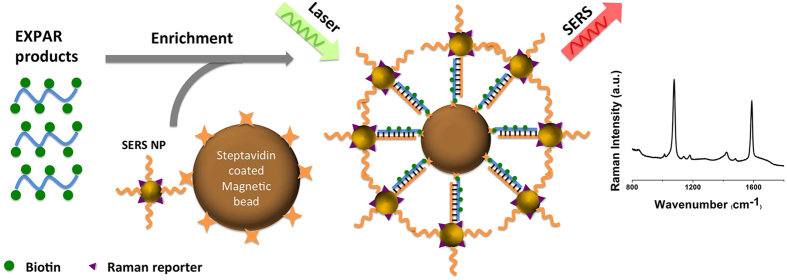
Schematic illustration of EXPAR-based SERS assay for target DNA detection.

The scheme of EXPAR-based SERS assay is illustrated in Fig. 5. EXPAR was first used to amplify the DNA target. During the amplification of DNA target by EXPAR, dUTPs with biotin tags were randomly incorporated onto the amplified DNA targets. Biotinylated EXPAR products were then enriched with streptavidin coated magnetic beads (MBs) followed by a wash to remove excess EXPAR products. Then, SERS nanoparticles (NPs) functionalized with Raman reporters (mercaptobenzoic acid-MBA in this case) and DNA probes (sequence shown in Table 2) on the surface were allowed to hybridize to the complementary EXPAR targets captured on the MBs followed by a wash to remove excess SERS NPs. SERS signals were then acquired on a Raman microscope where the intensity of a Raman peak at 1076 cm^−1^ (from the phenyl ring vibration modes of MBA) represented the amount of targets in the sample^37^.

**Figure 6:**
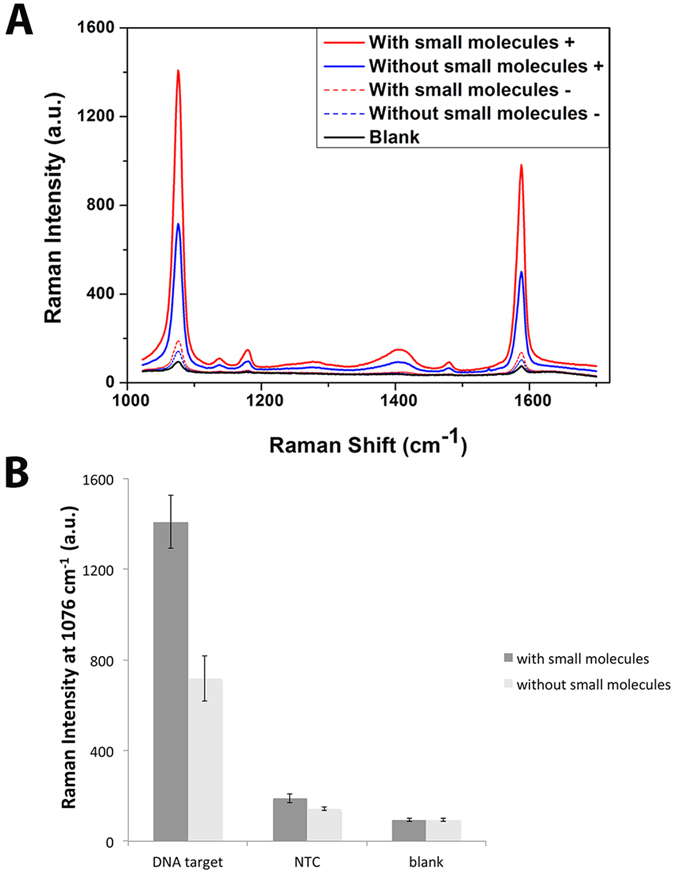
Effects of the combination of trehalose and TMAC on SERS. (**A**) SERS spectra with DNA target with small molecules, DNA target without small molecules, NTC with small molecules, NTC without small molecules, blank (no EXPAR product control). (**B**) Bar graph of average Raman intensities at 1076 cm-1 with and without trehalose and TMAC. Error bars represent Standard Error (SE), n = 3.

Figure 6 shows the comparison of assay performance with and without additives. Briefly, the addition of trehalose and TMAC resulted in an increased signal for the positive sample while supressing non-specific amplifications. This thus suggested that including additives may enhance overall EXPAR assay performance. TMAC was chosen over the other protein-based additives because chemical-based strategies were potentially of lower running cost.

We further tested the effects of the additives on EXPAR with complex biological samples. The limit of detection with or without trehalose and TMAC was tested by titrating target into a background of cell line derived RNA. As shown in Supplementary Figure S9, the limit of detection was improved by 10-fold with the addition of trehalose and TMAC, which demonstrated that the additives could be useful in improving EXPAR sensitivity, even in complex samples.

To illustrate the generality of including additives to isothermal amplification, the effect of one of the additives studied, i.e. SSB proteins, on Loop Mediated Isothermal Amplification (LAMP) was investigated^38^. We found that the inclusion of SSB proteins improved the sensitivity of LAMP by 10-fold (see Supplementary Figure S10). Apart from LAMP, we also note that small molecules such as trehalose and SSB proteins are key components of another isothermal Recombinase Polymerase Amplification (RPA)^39^,^40^, which further supports that the inclusion of additives (and their combinations) are generally useful for improving isothermal amplification strategies.

While we have not provided an exhaustive list of additive combinations, we believe that the type(s) of additives used would have to be dictated by the objective and readout method of the application. For example, an EXPAR application based on real-time measurements would benefit more from additives such as TMAC, betaine and SSB proteins to delay the onset of background amplification, which in turn may improve assay sensitivity.

In conclusion, we demonstrated the use of several small molecules including trehalose, TMAC, BSA and SSB proteins, to enhance EXPAR. The addition of trehalose in EXPAR was found to increase the yield, whereas the addition of TMAC, BSA and SSB proteins were found to increase the specificity of EXPAR. Depending on the nature of the experiment, these small molecules may be employed to increase the efficiency and specificity of EXPAR. Lastly, combinations of additives can also synergistically enhance EXPAR assay performance. We believe the information provided in this study can serve as a reference that will benefit the wider isothermal amplification community.

## Methods

### Reagents and Materials

EXPAR templates, DNA targets and DNA probe functionalized on AuNPs were purchased from Integrated DNA Technologies. The sequences of DNA used are shown in Table 2. The Vent (exo-) polymerase, Nt.BtsNBI nicking enzyme, the ThermoPol buffer, the NEBuffer 3.1, BSA, SSB proteins, dNTPs and the Streptavidin magnetic beads, were purchased from New England BioLabs. The Biotin-11-dUTP was obtained from Biotium. Ethylene glycol, propylene glycol, betaine, DMSO, trehalose, TMAC, and HAuCl_4_, sodium citrate dehydrate, 4-mercaptobenzoic acid (MBA) were purchased from Sigma-Aldrich.

### Standard EXPAR reactions

EXPAR was prepared similarly to previously reported protocols^5^,^7^. Briefly, each EXPAR was separated as part A and part B. Part A consisted of 150 nM X’-X’ template, 250 μM deoxynucleotide triphosphates (dNTPs), 0.5x Nt.BstNBI buffer (50 mM NaCl, 25 mM Tris-HCl, 5 mM MgCl_2_, 50 μg/mL BSA, pH 7.9) and 10 nM target DNA. Part B consisted of 0.4 U μL^−1^ Nt.BstNBI nicking enzyme, 0.05 U μL^−1^ Vent (exo-) DNA polymerase, 1x ThermoPol reaction buffer (20 mM Tris-HCl, 10 mM (NH_4_,)_2_SO_4_, 10 mM KCl, 2 mM MgSO_4_, 0.1% Triton X-100, pH 8.8), 2.5 nM of SYTO 9 and Ultrapure Water. The two parts were prepared at 4 °C and mixed immediately before placing in a qPCR machine. The reaction was performed in a volume of 10 μL at 60 °C for 20 min.

The amounts included in EXPAR experiments are as stated in the text. For the combination of trehalose and TMAC, the concentrations of trehalose and TMAC used were the concentrations that gave the best outcomes according to the experiments investigating the effects of the individual small molecule.

### Real-time fluorescence detection

Fluorescence intensity was monitored in real-time at 30 s intervals for 20 min using an ABI 7500 qPCR machine.

### Gel electrophoresis

10 μL of EXPAR products were mixed with 1 μL of gel loading dye and analysed with agarose gel stained with GelRed. 1.5% agarose gel was run at room temperature in 1x SB buffer at 250 V for 10 min. The gel was imaged by an ultraviolet (UV) transilluminator. Band intensities in the gel were analysed using the densitometry module in ImageJ software. The band intensities from the two sets of data were normalized to the average band intensity of the no-target control (NTC) without small molecules.

### Preparation of SERS nanoparticles (SERS NPs)

SERS NPs were prepared according to a previous study^37^. Briefly, gold nanoparticles were synthesized by citrate reduction of HAuCl_4_^41^. Gold nanoparticles (AuNPs) with a diameter of 60 nm were used in this project. 100 μM Thiolated DNA was first activated by 10 mM TCEP to obtain 50 μM activated thiolated DNA oligonucleotides (IDT). 1.5 mL of SERS NPs were concentrated into 1 mL and mixed with 10 μL 50 μM thiolated DNA at RT for 6 hours. Raman reporters 4-mercaptobenzoic acid (MBA) were then added to the AuNPs and incubated at RT overnight. Next, 0.6 M NaCl in 1 mM PBST buffer was used to age the SERS NPs at RT. SERS NPs were centrifuged and re-dispersed into 160 μL Ultrapure water (concentration of SERS NPs was therefore 0.5 nM) before use on the EXPAR-based SERS assay.

### EXPAR/SERS assay

Amplified DNA target was first incubated and captured using 5 μL of streptavidin coated MBs at RT for 15 min. MBs were then washed three times with 0.25x PBST buffer to remove other components from EXPAR. MBs were re-dispersed in the hybridization buffer (10 mM Tris-HCl, 10 mM EDTA and 50 mM NaCl) before the addition of 5 uL of 0.5 nM SERS NPs for hybridization. MBs with SERS NPs were incubated at 37 °C for 30 min. MBs were washed three times and re-dispersed in 0.25x PBST buffer for SERS detection by a portable IM-52 Raman Microscope with a 785 nm wavelength laser and 73 mW laser power. Three average spectra were obtained, each from 10 measurements with 2 s integration time.

## Additional Information

**How to cite this article**: Mok, E. *et al*. Comprehensive evaluation of molecular enhancers of the isothermal exponential amplification reaction. *Sci. Rep.* **6**, 37837; doi: 10.1038/srep37837 (2016).

**Publisher's note:** Springer Nature remains neutral with regard to jurisdictional claims in published maps and institutional affiliations.

## Supplementary Material

Supplementary Information

## Figures and Tables

**Table 1:** List of small molecules investigated in this study, their corresponding functions and their effects on EXPAR.

Small molecule	Mode of action	Effects on EXPAR			
		Yield		Specificity	
		dsDNA	ssDNA	dsDNA	ssDNA
Ethylene glycol	• Reduce DNA Tm^33^,^42^	No effect	− −	No effect	+
Propylene glycol		No effect	−	No effect	+
DMSO	• Disrupts base pairing – prevent intramolecular stem loops in GC-rich template^22^	No effect	− −	+	+++
Betaine	• Reduces DNA Tm^15^	− − −	− − −	++	+++
	• Isostabilizes DNA^15^				
	• Facilitates strand separation^43^				
Trehalose	• Reduces DNA Tm^13^	− −	+	−	− −
	• Thermostabilization of enzyme^19^				
TMAC	• Stabilizes A-T pairs^22^	−	−	+++	+++
	• Isostabilizes DNA^23^				
BSA	• Scavenges contamination^44^	No effect	−	− −	+++
	• Stabilizes DNA polymerase^45^				
SSB proteins	• Stabilizes single-stranded species^29^	−	No effect	++	+++

The positive (+) and negative (−) effects are indicated for each molecule. The effectiveness of each molecule is indicated by increasing number of (+) and (−).

**Table 2:** The sequences used in EXPAR and SERS assay are listed below (5′ - >3′).

DNA target (miR-200a)	TAACACTGTCTGGTAACGATGT
EXPAR template	ACATCGTTACCAGACAGTGTTActAACAGACTCtACATCGTTACCAGACAGTGTTAga-NH2
Probe functionalized on AuNPs	ACATCGTTACCAGACAGTGTTACTAACAGACTCTACATCGTTACCAGACAGTGTTAGATTTTTTTTTTTTTTT/3THIOMC3-D/
