# Supplementary material for: Comprehensive evaluation of molecular enhancers of the isothermal exponential amplification reaction
Source: Sci Rep. 2016 Dec 2;6:37837. doi: 10.1038/srep37837 (PMC5133538; doi:10.1038/srep37837)
Supplement: Supplementary Information [file srep37837-s1.pdf]

Electronic Supplementary Information

Comprehensive evaluation of molecular enhancers of the isothermal exponential amplification reaction

Ellie Mok,[ab] Eugene Wee,[a*] Yuling Wang[a*] and Matt Trau[ab*]

[a] *Centre for Personalized Nanomedicine, Australian Institute for Bioengineering and Nanotechnology (AIBN), Corner College and Cooper Roads (Bldg 75), Brisbane, QLD 4072, Australia.*

[b] *School of Chemistry and Molecular Biosciences, The University of Queensland, Brisbane, QLD 4072, Australia*

[*] *Corresponding: j.wee@uq.edu.au, y.wang27@uq.edu.au, m.trau@uq.edu.au*

Supplementary Figure S1

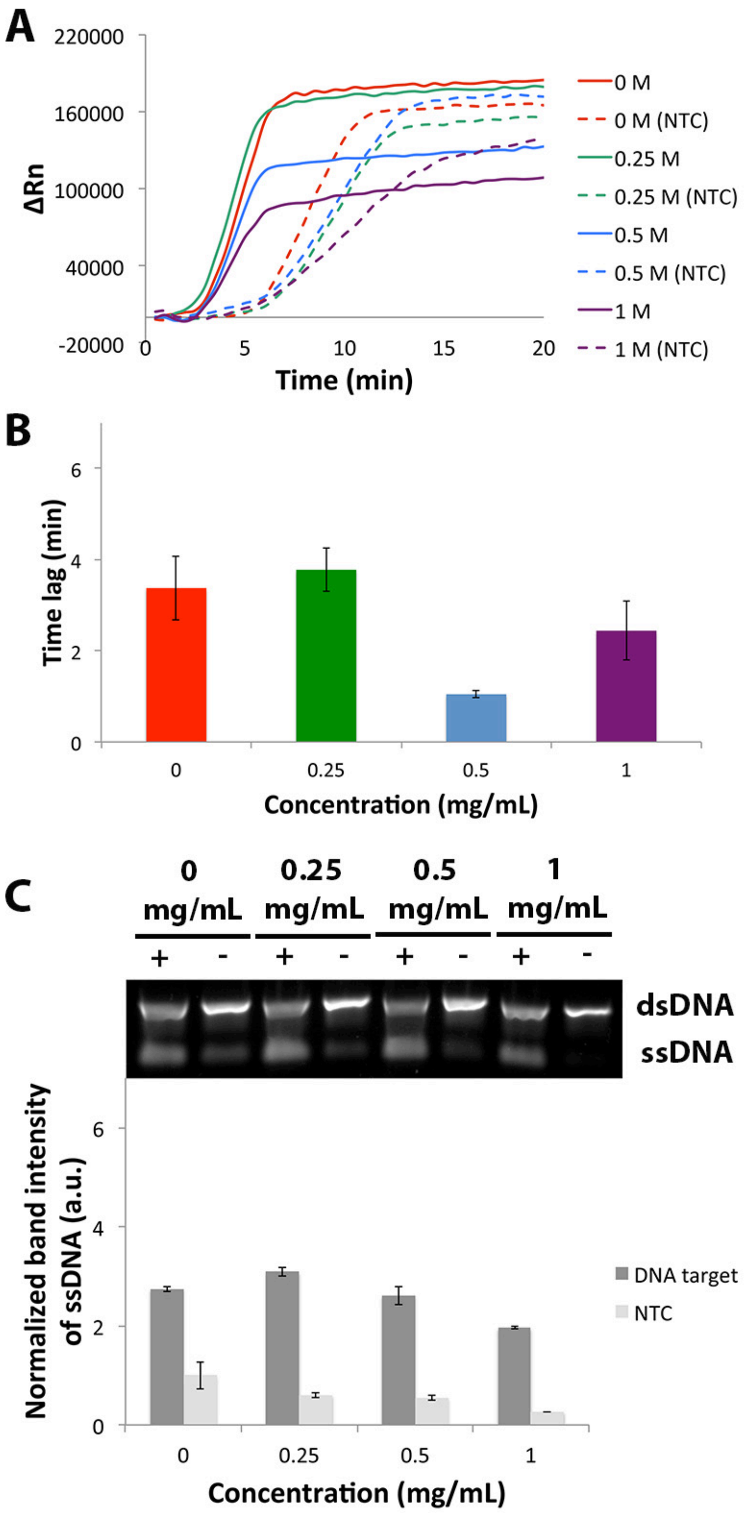

Fig. S1 Effects of different concentrations of BSA on EXPAR. A) Typical real-time amplification plot of EXPAR performed with 0, 0.25, 0.5 and 1 mg/mL of BSA. Solid lines: with target. Dotted lines: no target control (NTC). B) Time lag between targeted and NTC. C) Top: Typical gel electrophoresis image of corresponding EXPAR products. Bottom: Bar graph of average band intensities of ssDNA normalized to NTC without BSA. Error bars represent SD, *n*=2.

Supplementary Figure S2

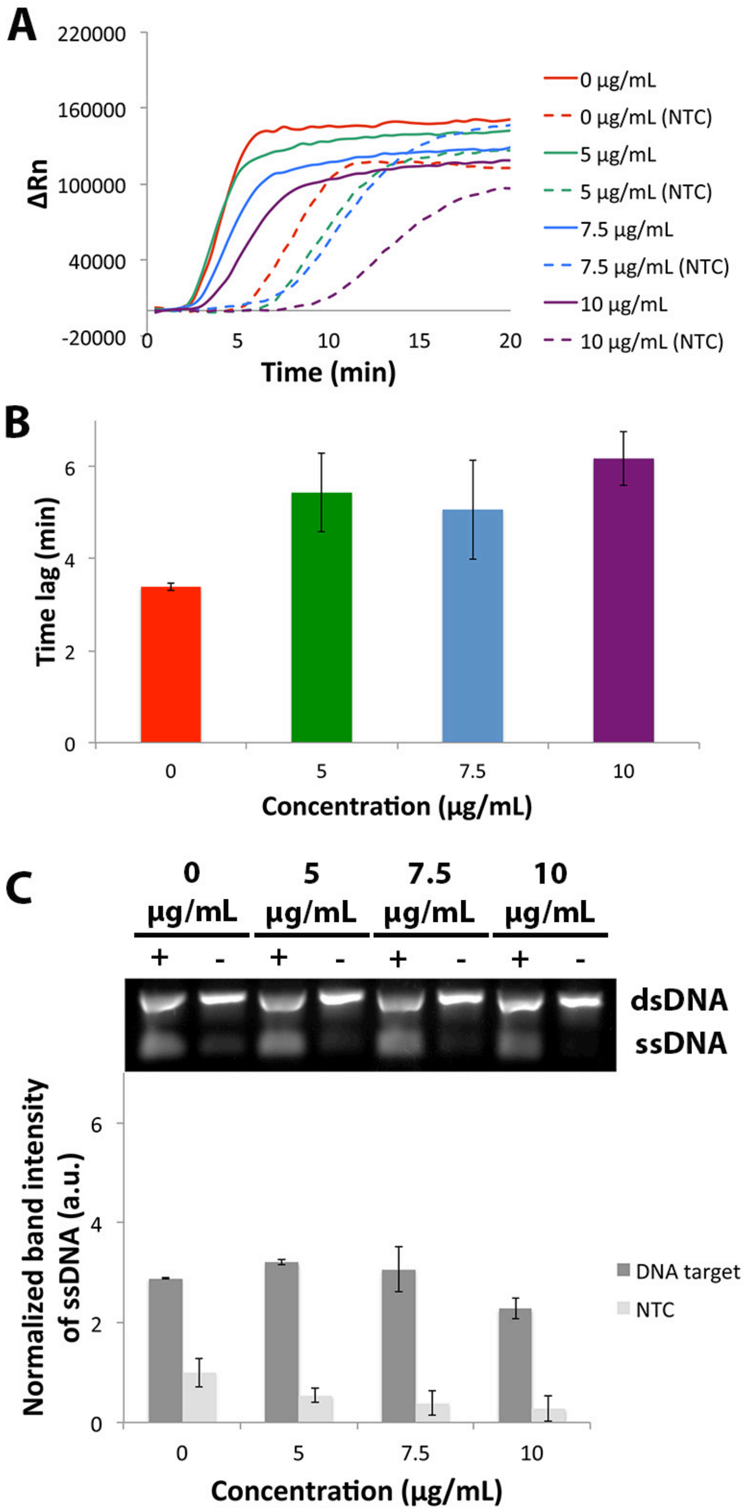

Fig. S2. Effects of different concentrations of SSB proteins on EXPAR. A) Typical real-time amplification plot of EXPAR performed with 0, 5, 7.5 and 10 µg/mL of SSB proteins. Solid lines: with target. Dotted lines: no target control (NTC). B) Time lag between targeted and NTC. C) Top: Typical gel electrophoresis image of corresponding EXPAR products. Bottom: Bar graph of average band intensities of ssDNA normalized to NTC without SSB proteins. Error bars represent SD, n=2.

Supplementary Figure S3

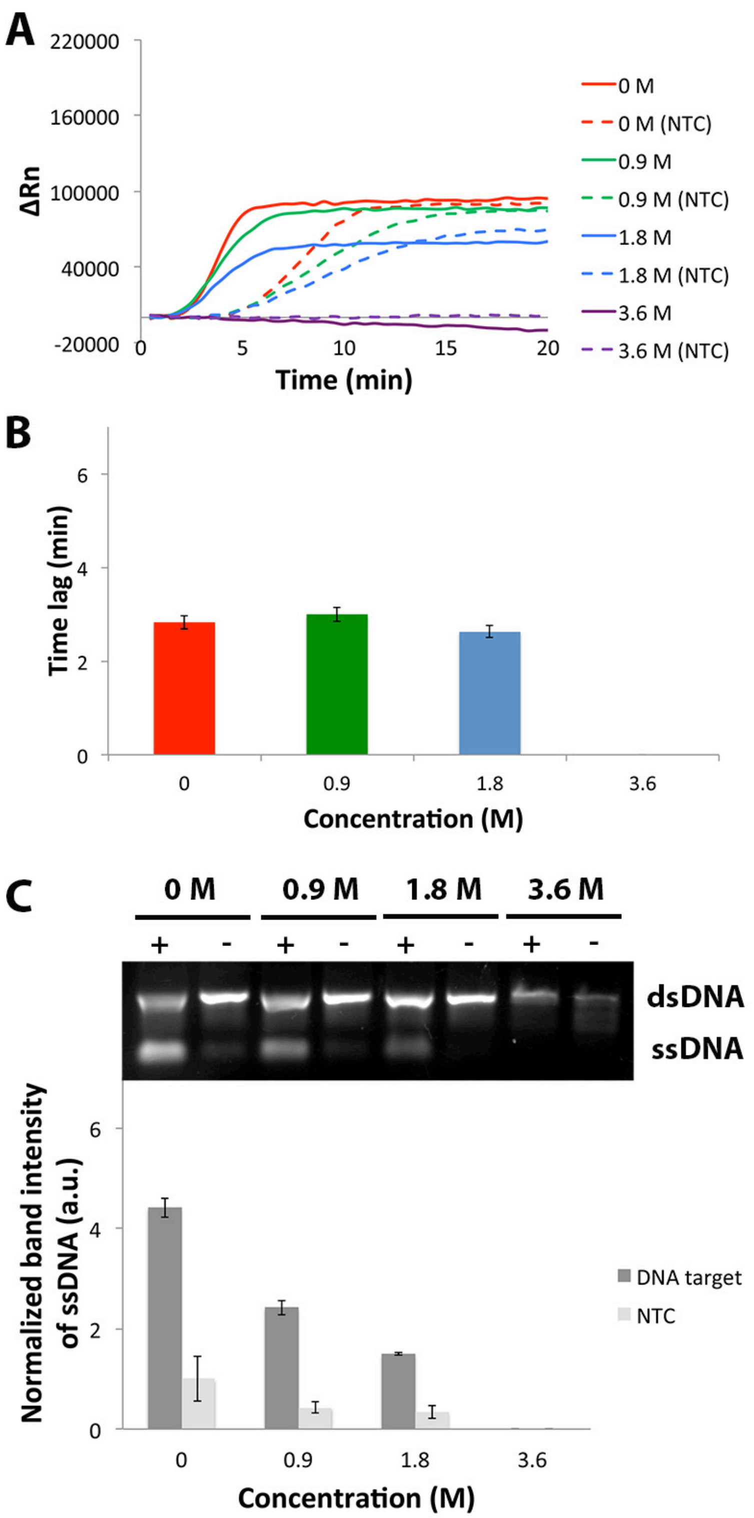

Fig. S3 Effects of different concentrations of ethylene glycol on EXPAR. A) Typical real-time amplification plot of EXPAR performed with 0, 0.9, 1.8 and 3.6M of ethylene glycol. Solid lines: with target. Dotted lines: no target control (NTC). B) Time lag between targeted and NTC. C) Top: Typical gel electrophoresis image of corresponding EXPAR products. Bottom: Bar graph of average band intensities of ssDNA normalized to NTC without ethylene glycol. Error bars represent SD, *n*=2.

Supplementary Figure S4

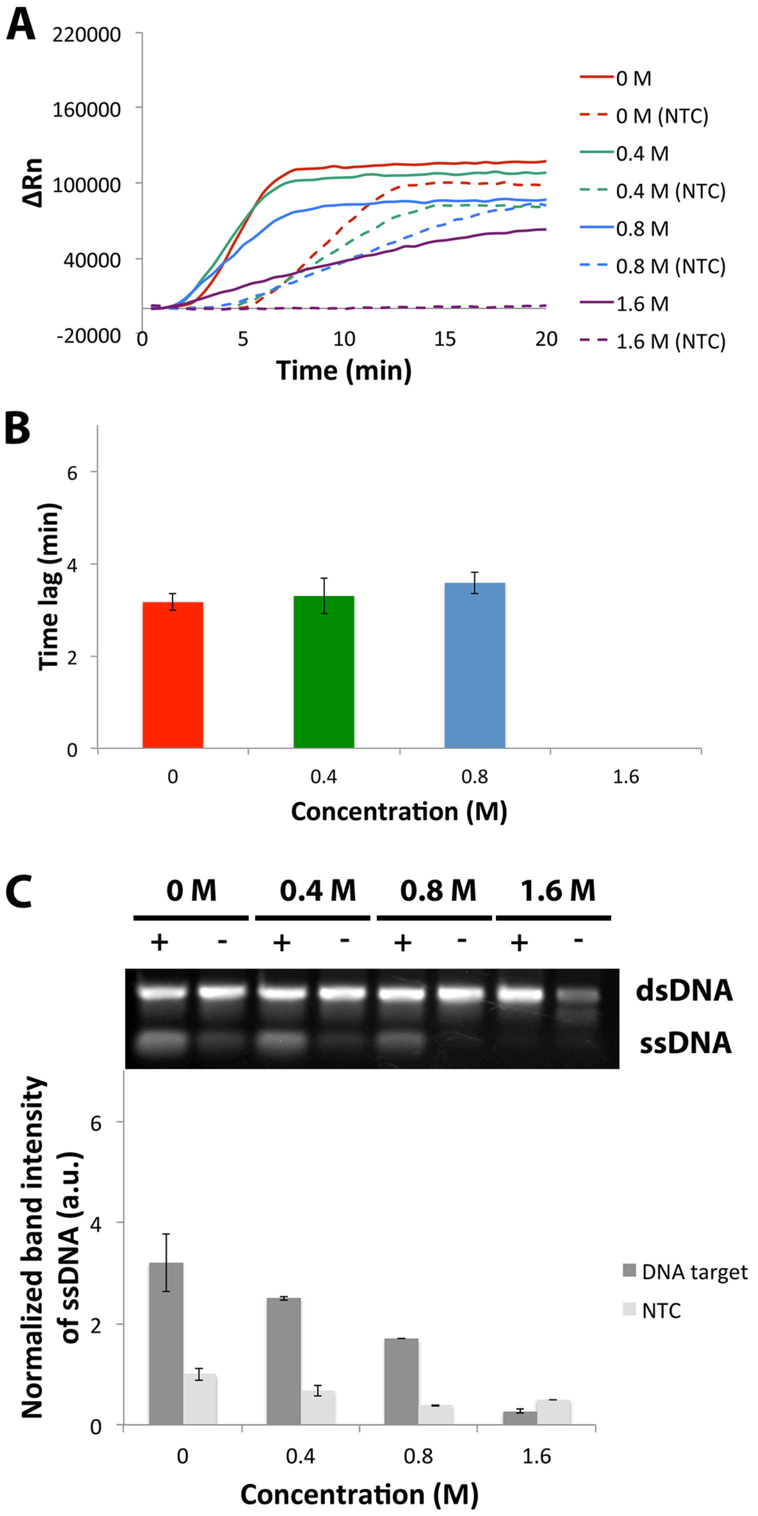

Fig. S4 Effects of different concentrations of propylene glycol on EXPAR. A) Typical real-time amplification plot of EXPAR performed with 0, 0.4, 0.8 and 1.6 M of propylene glycol. Solid lines: with target. Dotted lines: no target control (NTC). B) Time lag between targeted and NTC. C) Top: Typical gel electrophoresis image of corresponding EXPAR products. Bottom: Bar graph of average band intensities of ssDNA normalized to NTC without propylene glycol. Error bars represent SD, *n*=2.

Supplementary Figure S5

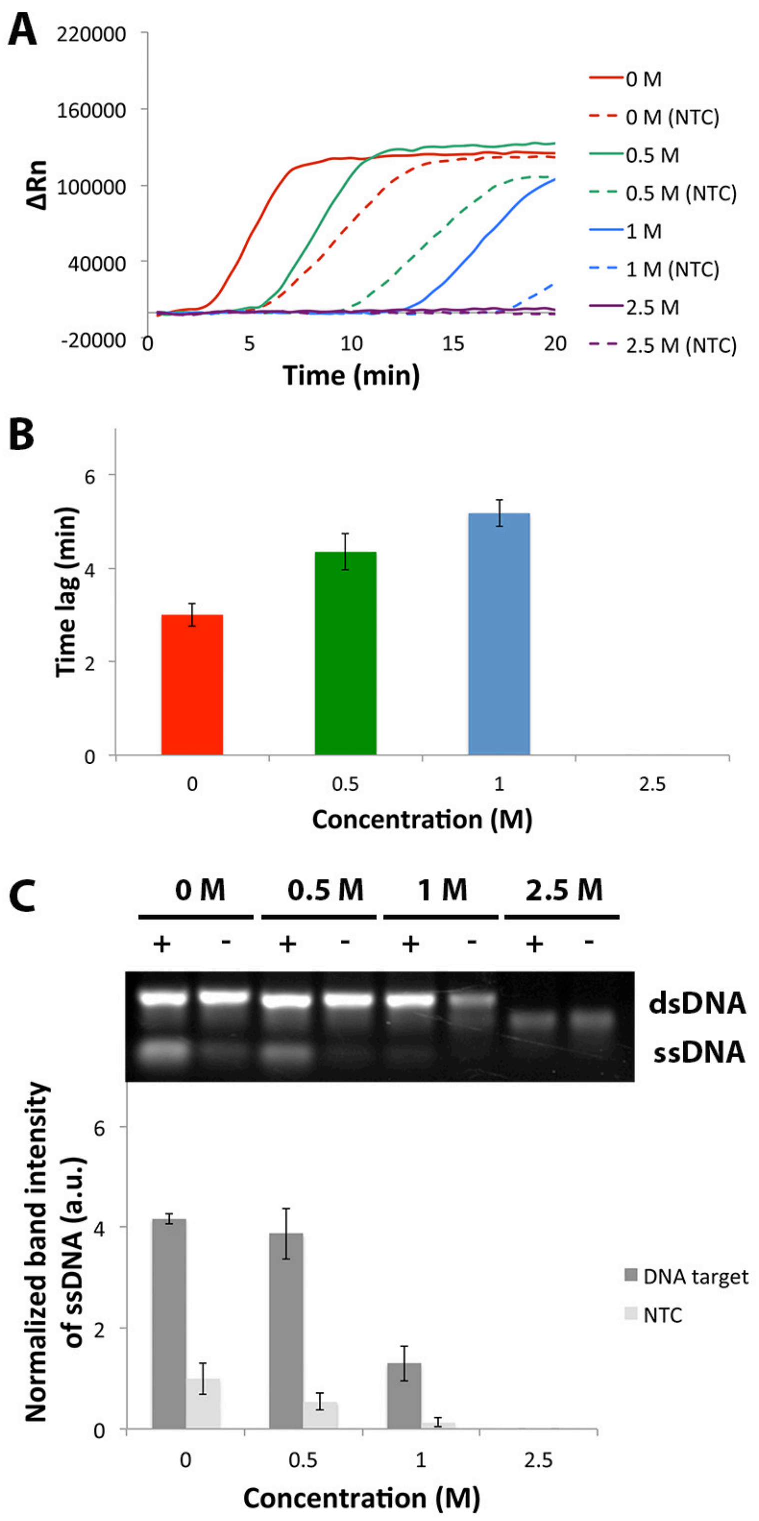

Fig. S5 Effects of different concentrations of betaine on EXPAR. A) Typical real-time amplification plot of EXPAR performed with 0, 0.5, 1 and 2.5 M of betaine. Solid lines: with target. Dotted lines: no target control (NTC). B) Time lag between targeted and NTC. C) Top: Typical gel electrophoresis image of corresponding EXPAR products. Bottom: Bar graph of average band intensities of ssDNA normalized to NTC without betaine. Error bars represent SD, n=2.

Supplementary Figure S6

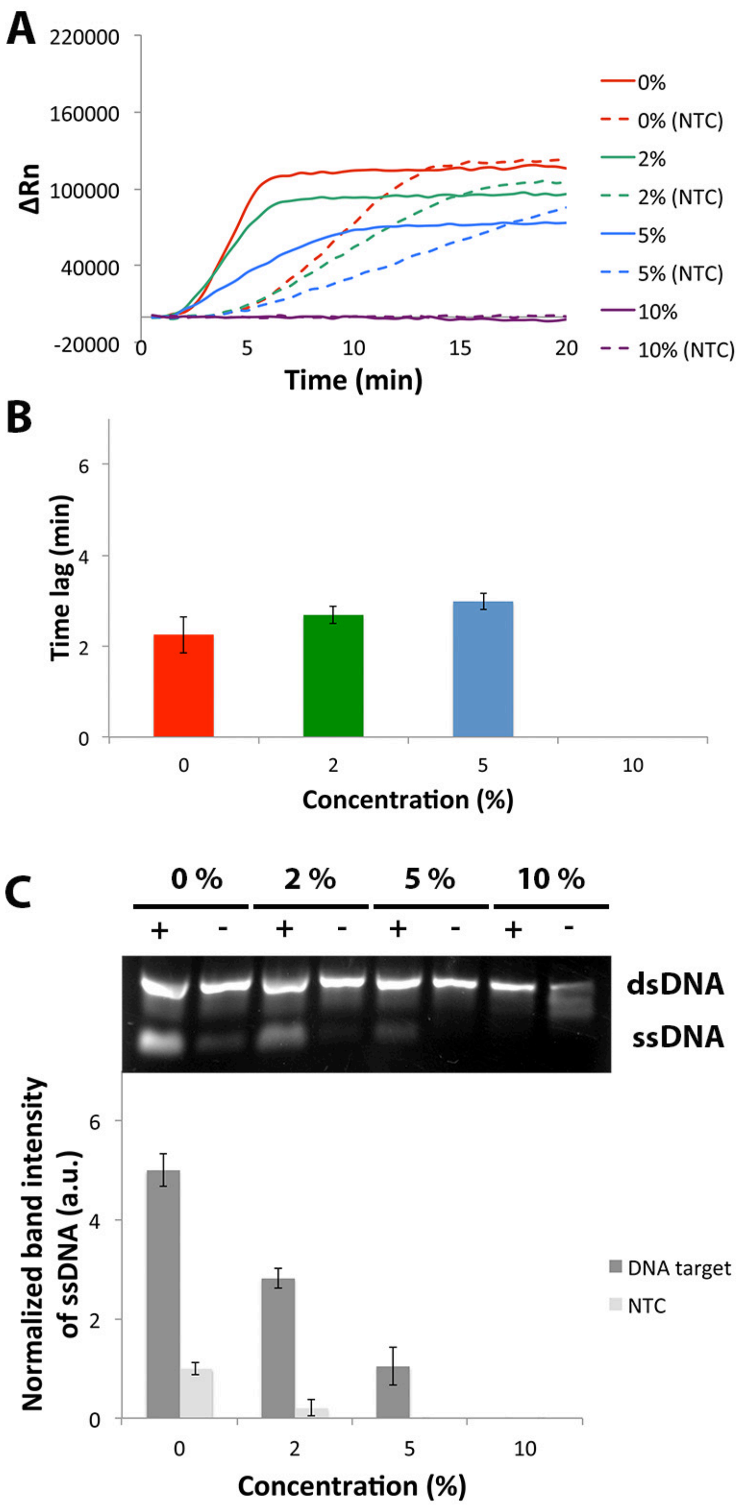

Fig. S6 Effects of different concentrations of DMSO on EXPAR. A) Typical real-time amplification plot of EXPAR performed with 0, 2, 5 and 10% of DMSO. Solid lines: with target. Dotted lines: no target control (NTC). B) Time lag between targeted and NTC. C) Top: Typical gel electrophoresis image of corresponding EXPAR products. Bottom: Bar graph of average band intensities of ssDNA normalized to NTC without DMSO. Error bars represent SD, *n*=2.

Supplementary Figure S7

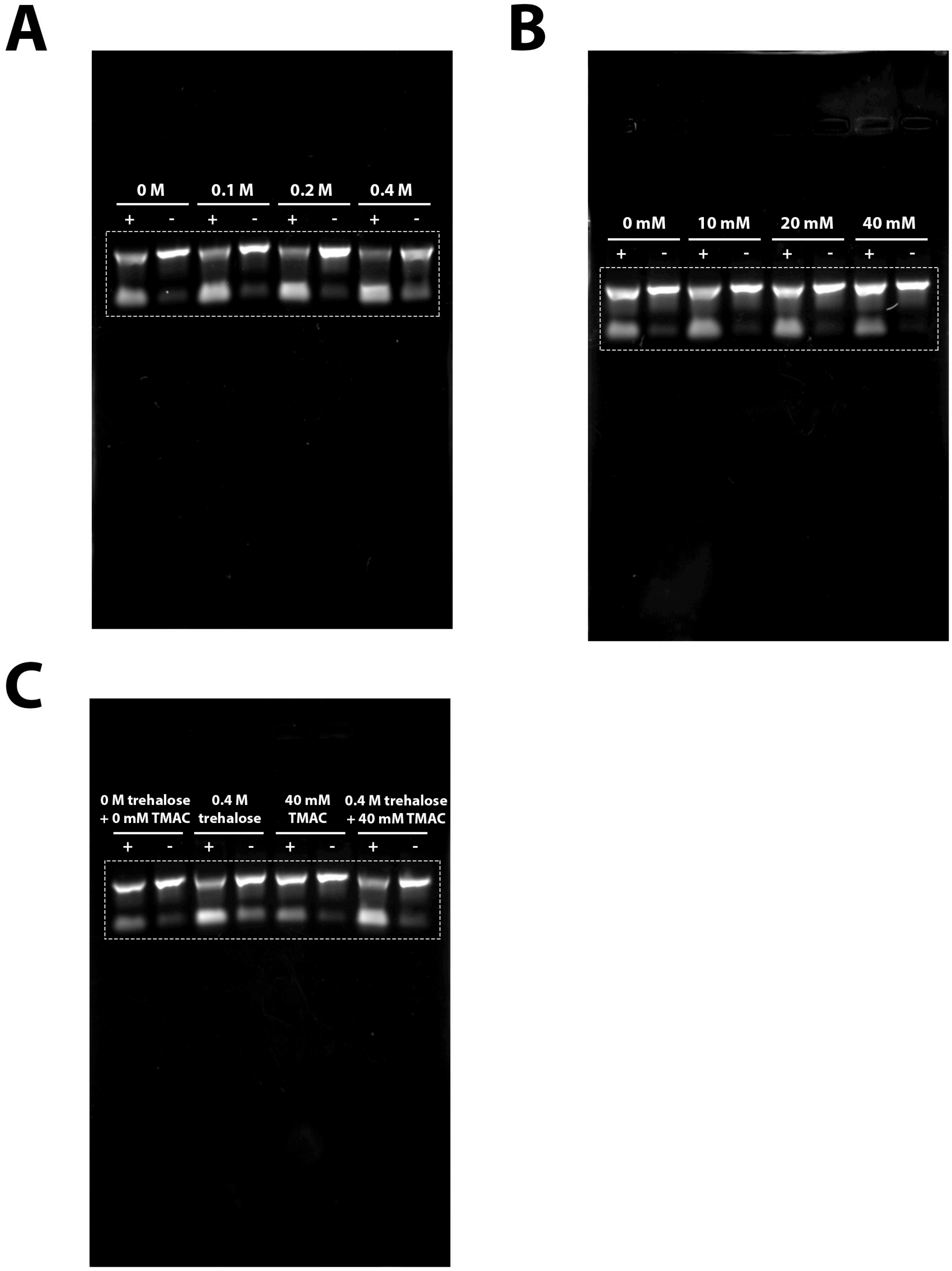

Fig. S7 Full-length gel images shown in main article. Effects of (A) trehalsoe, (B) TMAC, (C) combination of trehalose and TMAC on EXPAR. The dotted boxes represent the cropped regions shown in the Fig. 2, 3, 4 in the main article.

Supplementary Figure S8

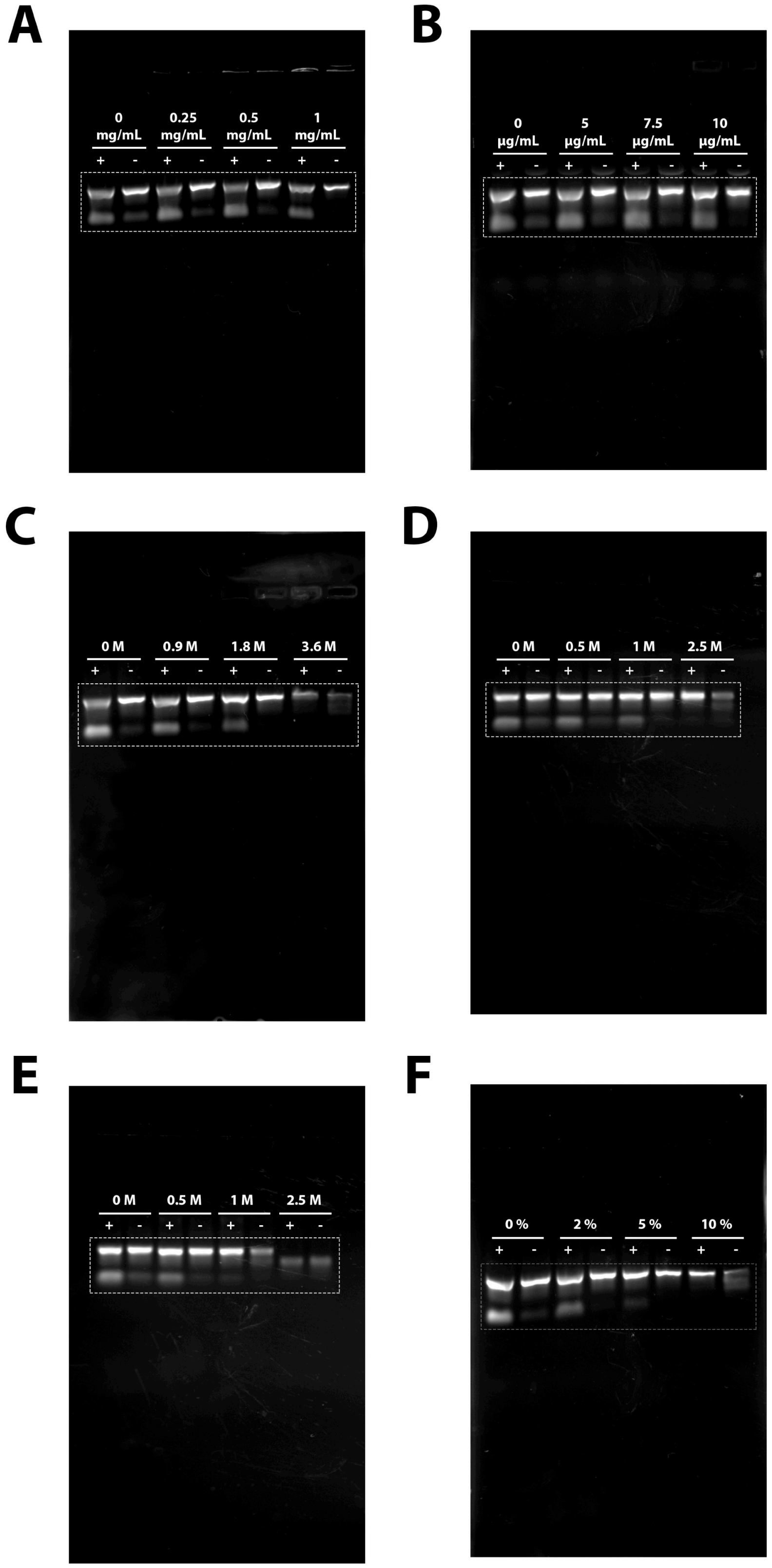

Fig. S8 Full-length gel images shown in electronic supplementary information. Effects of (A) BSA, (B) SSB proteins, (C) ethylene glycol, (D) propylene glycol, (E) betaine, (F) DMSO on EXPAR. The dotted boxes represent the cropped regions shown in the Fig. S1, S2, S3, S4, S5, S6 in the Supplementary information.

Supplementary Figure S9

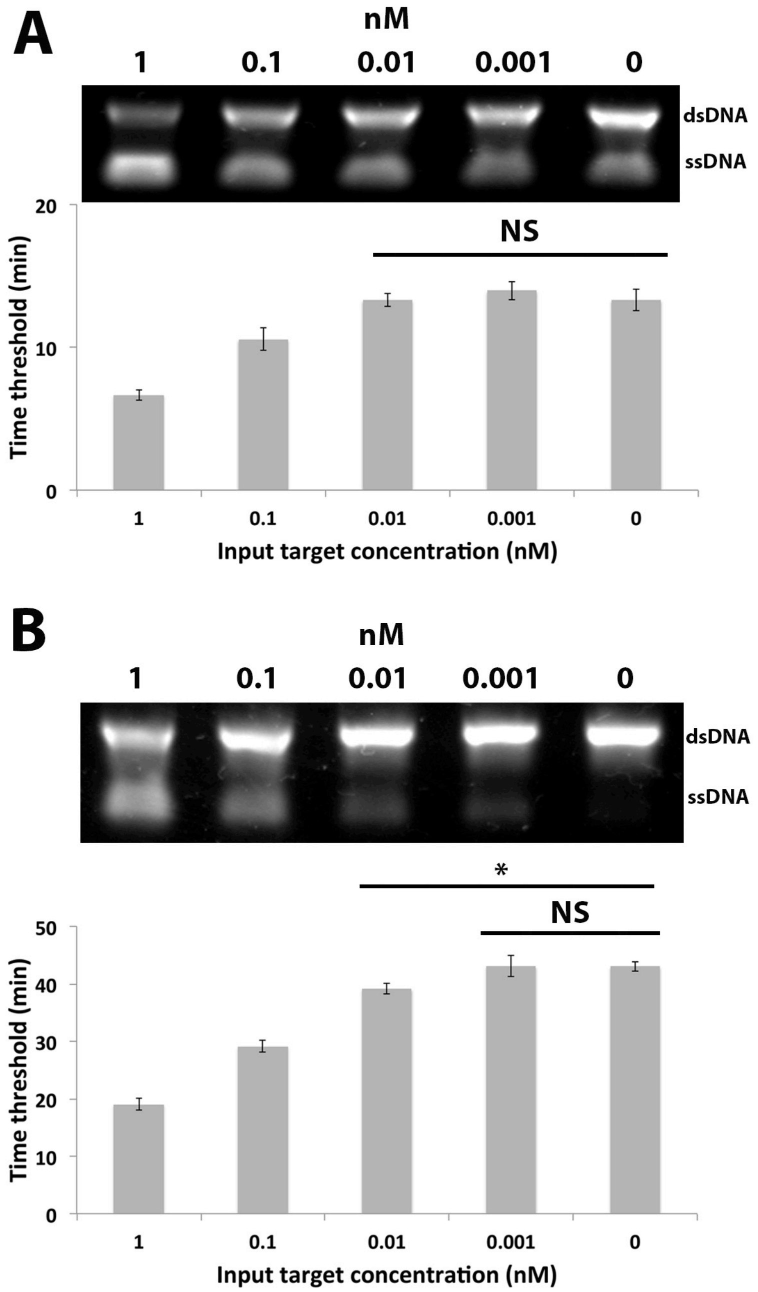

Fig. S9 Effects of the combination of trehalose and TMAC on the sensitivity of EXPAR. Standard EXPAR reactions with background 5 ng/µl MDA-MB-231 cell line and different input target concentrations. (A) Sensitivity assay without trehalose and TMAC. (B) Sensitivity assay with trehalose and TMAC. Top: Typical gel electrophoresis image of corresponding EXPAR products. Bottom: Bar graph of average time threshold of different input target concentrations. Error bars represent SD, n=2. * represents significant P value. NS: $P > 0.05$ (not significant), *: $P \leq 0.05$ (significant difference from no target control).

Supplementary Figure S10

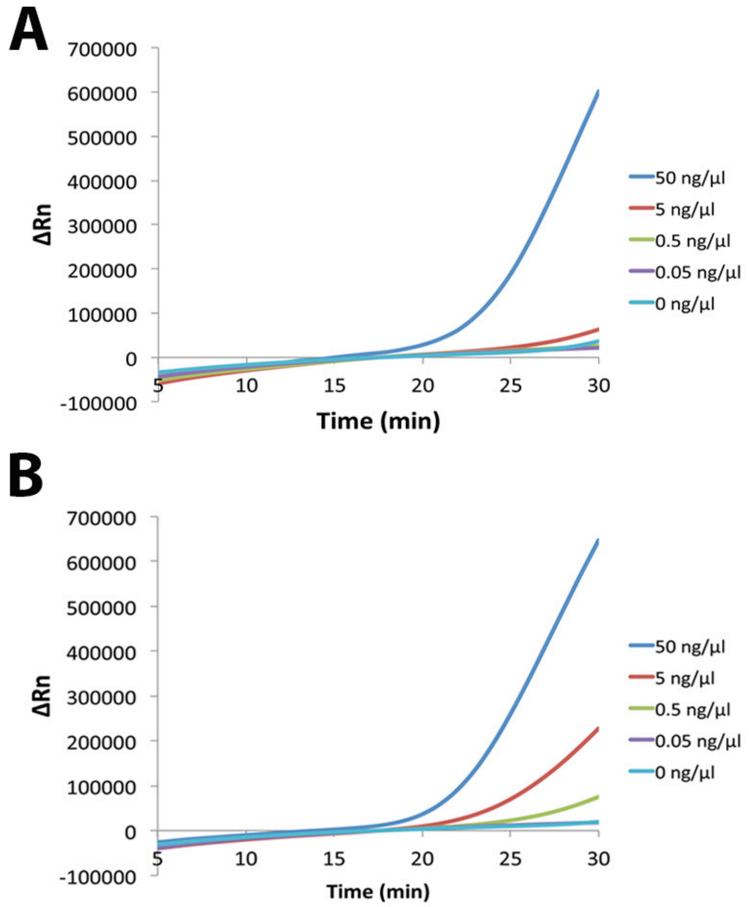

Fig. S10 Effects of SSB proteins on LAMP. Typical real-time amplification plot of LAMP performed (A) without SSB proteins (B) with 0.5 µg SSB proteins.

Supplementary Table S1

Effects of small molecules on the melting temperature (Tm) between target and template.

**Small molecule**	**Melting temperature (Tm) ± Standard deviation**
None	67.35 ± 0.07
1.8 M Ethylene glycol	62.61 ± 0.37
0.8 M Propylene glycol	63.93 ± 0.25
1 M Betaine	65.61 ± 0.36
5% DMSO	64.55 ± 0.37
0.4 M Trehalose	65.19 ± 0.04
40 mM TMAC	65.43 ± 0.12
1 mg/mL BSA	66.11 ± 0.59
10 µg/mL SSB proteins	66.92 ± 0.25

Standard EXPAR reactions were setup in the absence of enzymes. Reactions were run in qPCR machine with different temperatures and times setup: 95 °C for 30 sec, 25 °C for 10 min, increased to 80 °C with a rate of 1 °C per 30 sec.
